# An emphasis of T-cell subsets as regulators of periodontal health and disease

**Published:** 2021-09-20

**Authors:** Saranya Balaji, Priyanka K. Cholan, Dhayanand John Victor

**Affiliations:** Department of Periodontics, SRM Dental College, Chennai, Tamil Nadu, India

**Keywords:** T-cells, adaptive immunity, periodontal disease, T-cell subsets

## Abstract

**Background:**

The pathogenesis of complex diseases like periodontitis is moderated by the balance in immune inflammatory responses. T-lymphocytes are immune cells that descend from the bone marrow. Furthermore, they develop in the thymus playing an indispensable role in adaptive immune responses. The periodontal microenvironment allows differentiation of various groups of T-lymphocytes such as CD4^+^ (Th1/Th2/Th17/Treg/Tfh/Th9/T22), CD8^+^ cells, gamma-delta (*γ*d) T cells, or memory cells based on the current regional cytokine milieu to secrete distinct cytokines and other molecules required for resolution of inflammation or result in progression of the disease based on interactions among various cells.

**Aim:**

The dynamism of T-lymphocytes in the immunopathogenesis of periodontal diseases resulting in tissue destruction is established but the mechanisms of immunoregulation that underpins periodontal disease progression are cumbersome. This review aims to understand the distinct types of T cells and their effector functions with their portrayal in periodontal disease.

**Relevance for Patients:**

This review gives valuable insights on the possibility of predicting periodontal disease progression, on the management and its prognosis by evaluating specific cytokines of destructive T-cell phenotype, and on the future perspectives of therapeutic modalities including ways of modulating host immune and inflammatory responses to establish periodontal homeostasis and areas of research.

## 1. Introduction

Periodontitis is a multifarious chronic inflammatory disease involving a convoluted interaction between periodontal pathogens and the host immunity with the environmental and genetic factors having an impact on it. The regulation of immune inflammatory mechanisms determines the level of offset in the intricate balance between tissue homeostasis and disease progression [1]. Therefore, mastering of how the inflammatory reactions and the host immune mechanisms are modulated is significant to appreciate the pathogenesis of complicated diseases, namely, periodontitis.

The diverse range of antigen-specific T lymphocytes that mature in the thymus enables the host to respond to the evolving bacterial challenge [2]. This comprehensive antigen diversity of the T cells is engineered through the structural amendments in “T-cell receptors (TCRs)” created during the DN2 and DN3 stages of development of T cells through somatic recombination of selected gene-encoded segments [3]. Specific signals which include interaction of T-cell receptor (TCR) with antigenic peptide on major histocompatibility complex (MHC) molecule (signal 1) followed by costimulation (signal 2) and clonal expansion by cytokines activity (signal 3) are imperative in the activation and differentiation of T cells into any of their subtypes such as, helper T (Th) cells, cytotoxic (CD8^+^) T cells, gamma-delta (*γ*δ) T cells, regulatory T cells (Tregs), and memory T cells.

A healthy periodontium involves a network of polymorphonuclear leukocytes (PMNs), macrophages, and dendritic cells (DC) apart from *γ*δ T cells in immune surveillance, Tregs maintaining homeostasis, and a balanced Th1-Th2 action. The inflammatory response to the microbial biofilm at the tooth-gingival interface is initially encountered by the PMNs and macrophages. When there is failure in attaining complete resolution of inflammation, the bacterial products further trigger the antigen-presenting cells (APCs) to engage with the undifferentiated T cells, prompting the activation and differentiation of distinct T-cell subsets. Initially, the pronounced Th1 response attempts to clear the infection by potentiating phagocytosis. In the case of a weak Th1 immune response, the inflammation persists and there is a shift toward a Th2 response favoring B-cell activation with subsequent antibody production and Th17 activity. While the chronic B-cell activity is known to promote pro-inflammatory cytokines, Th17 cells also intensify the inflammation. Concurrently, the presence of Treg cells inhibits the Th1, Th2, and Th17 responses as an attempt to restore periodontal homeostasis. However, when the inflammatory environment prevails, it endorses a marked increase in Th17, Th1, and Th2 activity that surpasses the protective responses and paves way to tissue destruction resulting in periodontal disease [2].

In essence, the functionally diversified T cells eradicate infected cells by producing cytotoxic molecules and stimulating macrophages, B-lymphocytes, and additional T cells through their effector cytokines. Following antigen clearance, nearly, all of the effector cells are subjected to apoptosis and the ones that sustain become memory T cells [2].

The crucial involvement of T cells in regulating the host defense responses has been for a long time observed that the early stage of periodontal disease as delineated by Gemmell *et al*. is similar to the delayed-type hypersensitivity reaction, while the stable form of disease activity is a T-cell-mediated response with the progressive established lesion predominated by B cells [4]. This review pinnacles the main play of different T cells in periodontal health and disease pathogenesis, further presenting valuable insights on the future perspectives and areas of research.

## 2. TCR Signaling Pathways and Activation of T-Cell

**Figure 1:**
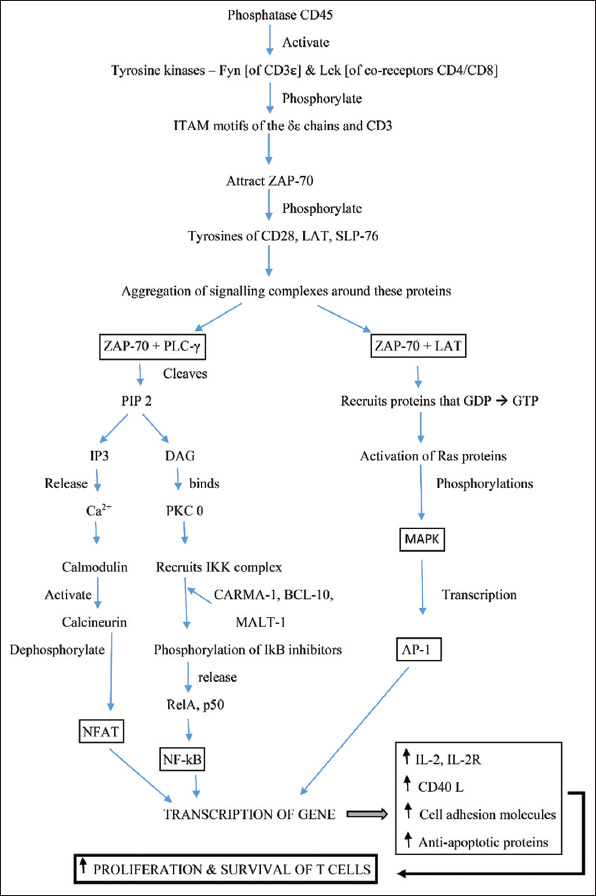
Signaling pathways in T-lymphocyte activation. LAT, linker of activated T cells; DAG, diacylglycerol; IP3, inositol triphosphate; PLC-*γ*, phosphoinositide phospholipase C; SLP-76, lymphocyte cytosolic protein; PKCθ, protein kinase C; RAS, family of GTP-binding proteins; LCK, lymphocyte-specific protein tyrosine kinase; FYN, member of Src tyrosine kinase; ZAP-70, ζ chain-associated protein kinase of 70 kDa.

In the lymph nodes, T cells bind with the DCs expressing antigen that is compatible to their specific TCR. A stable contact is established by exhibiting adhesion element ICAM-1, mature DCs, and highly antigenic ligands [5]. The CD4 and CD8 T cells identify antigens presented by MHC Class II and I molecules, respectively, while the *γ*δ T cells are not confined to MHC and recognize a wide variety of antigens. Following the capture of antigen by TCR, several “TCR microclusters” are formed to assist the rearrangement and induction of signaling molecules around the contact zone with DC forming the “immunological synapse,” which induces a nexus of signaling pathways on the inner aspect of the membrane and cytoplasm, culminating in the stimulation of the main transcription factors, such as nuclear factor kappa B, nuclear factor of activated T-cells, and activator protein 1 (Figure 1). To license the T-cell response toward an antigen and to complete the process of its activation, the second signal for costimulation is required. The costimulatory receptors and molecules, such as CD28, CD27, herpesvirus entry mediator, signaling lymphocyte activation molecule, and inducible T-cell costimulator (ICOS) potentiate synthesis of interleukin (IL) 2, which serves as an autocrine and paracrine growth factor. Further interactions induce antiapoptotic signals to prolong the life span of T-lymphocyte, stimulate activity of adhesion compounds, and increase production of cytokines and growth factors that promote T-cell proliferation and differentiation. The enhanced IL-2 levels stimulate clonal expansion of the corresponding T cells. A barricade to prevent T-cell overactivation is its expression of cytotoxic T-lymphocyte antigen 4 which overshadows CD28 for engaging with B7 proteins [6]. Markers of activation include CD69, CD71, CD25, and HLA-DR.

## 3. T-Cell Subsets

T-lymphocytes are categorized as helper cells (Th)/CD4^+^ cells, cytotoxic cells (Tc)/CD8^+^ cells, Tregs, and memory cells on the basis of functional differences [2]. The differentiation into any of these cell phenotypes is determined by the type of APC, activation state, nature and concentration of antigen, cytokine milieu, and presence of costimulatory molecules in the microenvironment.

### 3.1. Th cells

If the T-cell expresses CD4 surface molecule and is activated following antigen presentation by MHC Class II molecule, it differentiates into CD4^+^ T helper cell. Initially, Parish and Liew reported the existence of only two Th cell types, namely, Th1 and Th2 [7]. The individual cytokine characterizations of each Th cells were later outlined by Mossmann and Coffman [8]. Since then, Th1/Th2 paradigm was used in annotating the immunopathogenesis behind various chronic inflammatory disorders, including periodontitis. Later, it was known that T helper cells are more diversified and Th17 [9], Tregs [10], Th9 [11,12], and Th22 [13] were a new addition to the Th cell lineage. In addition, the property of plasticity in Th cells which enable transformation among subsets was demonstrated earlier.

#### 3.1.1. Th1/Th2 reciprocity

The Th1 differentiation is prompted by the cytokines IL-12, IFN-*γ*/interferon alpha (IFN-*α*), and IL-18 released from the activated DC or natural killer (NK) cells following the trigger by intracellular pathogens or lipid antigens [14]. These cytokines activate signal transducer and activator of transcription (STAT) 1 in the undifferentiated T cells which further stimulate the transcription factor T-box expressed in T-cell (T-bet), resulting in the induction of receptor IL-12R*β*2, thereby enabling these cells to be more amenable to IL-12. Furthermore, it activates STAT-4 which enhances IFN-*γ* secretion, the effector cytokine of Th1 cells. It also produces IL-2 and expresses CXC chemokine receptor (CXCR) 3 and CD161. During the later stages of evolution, expression of the receptor IL-18R*α* is increased, thereby permitting them to be sensitive to IL-18. The IL-12 activity is further potentiated by IL-18, resulting in a strong cell-mediated immune reaction against intracellular pathogens and the development of delayed hypersensitivity reaction [15,16].

In the diseased gingival tissues, Th1 cells are predominant and the resulting IFN-*γ* stimulates osteoclastogenesis, resulting in bone destruction through T-cell activation or recruitment of receptor activator of nuclear factor kappa-B ligand (RANKL) cells [2,17].

The cytokines IL-4, IL-33, and IL-11, in addition to IL-25, induce the differentiation of Th2 cells [18]. The transcription factor GATA-binding protein 3 (GATA-3; through activated STAT-6 and STAT-5) [19,20] is involved in the expression of effector cytokines and chemokines, including IL-4, IL-10, IL-13, IL-5, IL-9, IL-25, ICOS, and CC chemokine receptor (CCR) 4 [21,22]. These signaling molecules account for the anti-inflammatory action of Th2 cells and suppression of Th1 response, and are effective against extracellular pathogens. Some of the Th2 cytokines such as IL-4, IL-5, and IL-13 partake in immunoglobulin class switching in B cells [23]. With regard to periodontitis, Th2 cells hamper the secretion of matrix metalloproteinases (MMPs) and RANKL, thereby attenuating soft and hard tissue destruction. Periodontitis patients are noted to have reduced levels of IL-4.

An inhibitory effect is applied on the Th1 and Th2 cells through various processes. Their signature cytokines, IFN-*γ* and IL-4, suppress one another. Similar responses can also be seen in intracellular signals such as suppression of STAT-4 by GATA-3 and the resulting Th1 responses, whereas STAT-5 decreases expression of T-bet. GATA-3 expression can also be negatively regulated by T-bet itself [24,25].

#### 3.1.2. Th17/Treg axis

The Th1/Th2 paradigm ruled over the pathogenesis of various inflammatory and autoimmune disorders for several decades until a new subpopulation of Th cell called the Th17 cell was identified. Their differentiation is induced by the combined effect of IL-6 and/or IL-1 with transforming growth factor beta (TGF-*β*). IL-21 and IL-23 signals are essential for expanding and balancing the steady state of Th17 cells [26,27]. Although the Th17 signaling pathway is not well established, retinoic acid-related orphan receptors (ROR*γ*t) are presumed to be its master switch. The Th17 cells are known to primarily produce IL-17 (IL-17A and IL-17F), IL-21, and IL-22 [28]. IL-17A enhances C-X-C motif chemokine ligand 8 (CXCL8) expression and is essential for neutrophil recruitment. Th17 cells are known to defend the extracellular pathogens [28] and have pro-inflammatory as well as anti-inflammatory roles [29]. IL-17 stimulates epithelial cells, endothelial cells, and fibroblasts to release IL-6, prostaglandin E2 (PGE2) and IL-8, and promotes osteoblasts to produce RANKL; therefore, IL-17 is a prime influencer of periodontal bone resorption [30,31]. Gershon and Kondo found the functional phenotypic diversity of Tregs, which were named “suppressor cells” as they can suppress the activation and multiplication of T cells, thereby regulating inflammation [32]. Tregs were delineated as one of the functional Th cells by Sakaguchi *et al*. in 1995 [10] as they are forming 5–10% of Th cells in healthy individuals. Tregs require TGF-*β* and IL-2 for differentiation as well as cytokines such as IL-1 and IL-6 for inhibition, while the absence of IL-2 renders them anergic. The transcription factor Forkhead box protein P3 (FoxP3) is activated by TGF-*β*, thereby enabling the Tregs effector function in synthesizing TGF-*β*, IL-35, and IL-10. Based on origin and action, two Treg cell types have been observed, namely, natural cells (nTreg, CD4^+^, CD25^+^, and FoxP3^+^) of thymic origin and adaptive or induced regulatory cells (Tr1, Th3 CD4^+^, CD25^-^, and FoxP3^-^), which differentiate in the periphery, expose to IL-10 and TGF-*β*, and interact with immature DC [33,34]. Lately, a new subpopulation of induced Tregs called iTr35 cells, which mediates immunosuppression mainly by the production of IL-35, was described. In general, Tregs are known to suppress the pro-inflammatory environment of periodontal disease by promoting resolution of inflammation through IL-10 and TGF-*β*. It also prevents autoimmunity by tolerogenic responses (especially Tr1) [35].

Similar to the Th1/Th2 profile plasticity, counteracting processes in the local microenvironment promote the Th17/Treg cell responses. The main requisite for the evolution of Th17 as well as Treg cells is TGF-*β*. Activated Th cells express both the ROR*γ*t and FoxP3, but FoxP3 antagonizes the function of ROR*γ*t. The ROR*γ*t is downregulated by TGF-*β*, along with enhanced levels of IL-1 and IL-6 (e.g., in progressive periodontal lesions), whereas FoxP3 expression is upregulated. Conversely, in the absence of pro-inflammatory cytokines together with enhanced TGF-*β* expression, FoxP3 expression is advocated resulting in the development of Tregs (e.g., in stable periodontal lesion). During inflammation, the levels of retinoic acid is decreased, resulting in the outcome of reduced Treg cell differentiation as its transcription factor is related to retinoic acid, and this intervenes the balance shift towards a Th17 response [36]. Experimental evidence for this fact is currently not available.

#### 3.1.3. T follicular helper (Tfh) cells

The Tfh cells are known to assist the B cells in secreting antibodies. The development depends on the IL-6, IL-21, and IL-12. The persistent appearance of CXCR-5 together with mislaying of CCR-7 typifies Tfh cells which let them shift toward B-cell follicles manifesting CXCL13, where they initiate formation of germinal center, plasma cells, synthesis of memory type B cells, and secretion of various isotopic antibodies through their effector cytokines IL-10, IL-4, and IL-21 whose synthesis is induced by the transcription factor B-cell lymphoma 6 protein [37].

#### 3.1.4. Th9 cells

Despite the contradictory findings on the existence of these cells, IL-4 and TGF-*β* have been reported for their involvement in inducing cell differentiation. The Th9 cells secrete IL-9 and IL-10 while their actions are inhibited by IFN-*γ*. Further, the transcription factor PU.1 and JAK-STAT pathway was found to be involved in Th9 signaling responses. IL-9 is known to promote mast cell evolution and synthesis of IL-1*β*, IL-13, IL-6, and TGF-*β* [11,12]. Despite the contradicting protective and destructive roles assigned to Th9 cells in periapical and pre-malignant lesions, their activities in periodontal disease have not yet been identified.

#### 3.1.5. Th22 cells

The differentiation of Th22 cells is induced by IL-6, tumor necrosis factor alpha (TNF-*α*), and aryl hydrocarbon receptor. RAR-related orphan receptor C is known to initiate the Th22 cell signaling to produce IL-22 and induce lower levels of IL-17 and IFN-*γ* [13]. Their exact roles in periodontal disease are yet to be completely elucidated. However, these cells produce defensins in the epithelium, suggesting a role in the defense mechanism of gingival epithelium. On the other hand, increased level of IL-22 from Th22 cells has also been associated with osteoclastic activity and periodontal disease severity [38].

### 3.2. CD8^+^ T cells

If T cells express CD8 surface molecule and are activated following antigen presentation through MHC Class I molecule, T cells evolve into cytotoxic CD8^+^ T cells. Based on their function, activated T cells are grouped as cytotoxic T lymphocytes (Tc) and CD8^+^ regulatory T lymphocytes (CD8^+^ Tregs). The Tc cells activated through cytokines TNF-*α* and IFN-*γ* can attack and destroy virus-infected or tumor cells directly [39]. It stimulates cell death in focused cells through cytolytic granules or expression of Fas ligands [40]. The pore-forming protein (e.g., perforins), proteases (e.g., granzyme), fragmentin, and granulolysin in the cytolytic granules take part in the degradation of membrane lipids of target cells and favor DNA fragmentation [41]. The fact that CD8^+^ T cells can subdue other immune cells was reported by Gershon and Kondo in 1970 [32]. Within the CD8^+^ Treg cells, various subpopulations have been identified and found to produce regulatory mechanisms through several modes of actions, such as secretion of cytokines TGF-*β* and IL-10, direct binding of target cells, and induction of anergy in APCs [42].

### 3.3. Memory T cells

Memory T cells are long-lived cells, expressing quick mounting ability and strong immune response against their cognate antigen upon reexposure. CD4^+^ or CD8^+^ cell types are two possible forms of memory T cells, expressing CD45RO and lacking CD45RA [43]. There are various types of memory T cells, among which gingival-resident and circulating memory cells have been well documented in periodontal diseases [44,45].

### 3.4. NK T cells

They are distinctive type of T lymphocytes with the expression of uniform TCR *α* chain V*α*24-J*α*Q capable of recognizing glycolipid antigens exhibited by MHC-like CD1d molecules. NKT cells are suggested to demonstrate a modulatory function in periodontitis. Autoimmunity has also been propounded for its involvement in periodontal diseases. In addition, cross-reactivity of heat shock protein 60 (HSP60) and P.g GroEL has been reported in periodontitis. NKT cells can play key roles in combating such autoimmune responses through rapid secretion of IL-4 and IFN-*γ* [46].

### 3.5. γδ T cells

*γ*δ T cells form a small subpopulation of T lymphocytes (2-5%) in blood and are mostly present in the gut mucosa. They contain *γ*δ TCR which allow them to recognize whole antigens and respond to phosphoantigens instantly. Except producing IL-17 in the periodontium, these cells play role in the barrier surveillance where they produce amphiregulin to maintain tissue homeostasis [47].

## 4. T Cells in Periodontal Health

The defense system patrols the periodontium proactively to maintain homeostasis. Healthy gingiva comprises a rich inflammatory infiltrate dominated by T cells along with a network of DC and macrophages acting as APCs which organize local immunity while the B lymphocytes and plasma cells are scarcely found. PMNs that can interact with adaptive immune cells are present in healthy periodontium but in reduced levels compared to gingivitis and periodontitis lesions. Among the T-lymphocytes, CD4^+^ T helper cells are predominantly seen in healthy gingiva accompanied by Tc cells besides a minor proportion of *γ*δ T cells [48]. Gingival CD8^+^ T cells (CD8^+^ Tregs) possess regulatory actions essential for maintaining integrity of the tissue by repressing inflammation. They release TGF-*β* and IL-10, thereby promoting resolution and preventing bone destruction in periodontal diseases through the inhibition of osteoclastogenesis [49]. In the epithelial tissues, resident *γ*δ T cells form the majority of T-lymphocyte proportion and carry out immune surveillance at the barrier and help in maintaining tissue homeostasis through IL-17 secretion and epithelial repair to some extent by amphiregulin synthesis [47]. Wilharm *et al*. demonstrated the upregulation of gingival inflammation and prevalence of dysbiotic environment on the elimination of *γ*δ T cells [50]. The Th1 cells known to be effective against intracellular pathogens produce IFN-*γ* which would elicit a robust innate immune reaction and contain the infection by potentiating the phagocytic capacity of PMNs and macrophages [4]. Contrarily, bone resorption as a result of IFN-*γ*-induced osteoclastogenesis has also been noted, although few studies report the contradictory findings [2,17]. The Th2 cells known to be effective against extracellular pathogens mainly produce IL-4. Despite the protective role of Th2 cytokines in hampering periodontal destruction by diminishing MMP and RANKL secretion, they are also associated with B cells and found in progressive periodontal lesions [51]. The Th1/Th2 paradigm had several controversies where Th1 cytokines were suggested to be involved in gingivitis (stable lesion) and Th2 response in periodontitis (progressive lesion) by Seymour *et al*. [52]. Nonetheless, Ebersole and Taubman in 1994 proposed an opposite model. A Th1/Th2 framework proposed by Shapira *et al*. deciphers the spasmodic fashion of periodontal tissue destruction under a protective antibody response, according to which periodontal destruction relies on the homeostasis among type 1 and 2 responses in the local micro-environment and any switch in this homeostasis amongst the protective T helper type 2-dominant response and destructive T helper type 1-dominant response might act as a risk factor for disease initiation and progression [53,54]. The Th1/Th2 paradigm has been contradictory since its establishment in the late 1980s as it is based solely on a subcategory of clinical and empirical data. The differentiation of T cells that promotes tissue destruction not only occurs when there is a microbial insult but also in clinically healthy gingival conditions. According to the findings of an animal study, as a result of masticatory forces, the epithelial cells enhanced IL-6 expression to catalyze the differentiation of Th17 cells which supports the barrier protective role of Th17 cells in addition to their bone destructive property [55]. Tregs form up to 15% of Th cells and are vital for periodontal homeostasis. Even during the presence of local inflammation, they are associated with homeostasis in the osteoimmunology compartment which might be correlated to the stable lesion of gingivitis in few individuals. Further, Tregs inhibit Th1, Th2, and Th17 immune responses through regulatory cytokines IL-10 and TGF-*β* [34,56]. They do not secrete IL-2 but exhibit more CD25 and create a challenge for other effector T cells which require IL-2 leading to disruption of proliferative signals and a state of famine of other effector cells [57]. It also instigates apoptosis of other triggered effector T cells, thereby conferring tissue homeostasis by preventing tissue damage induced by the overactivated T cells [58,59]. According to Dutzan *et al.*, tissue-resident effector memory T cells (CCR7^-^ CD69^+^), which form the largest portion of T cells at the gingival barrier, endorses the function of memory cells at barrier sites in eliciting a rapid defense mechanism on the reexposure to cognate antigens, thereby preventing the ingress of bacterial toxins into the underlying connective tissue [48].

**Figure 2:**
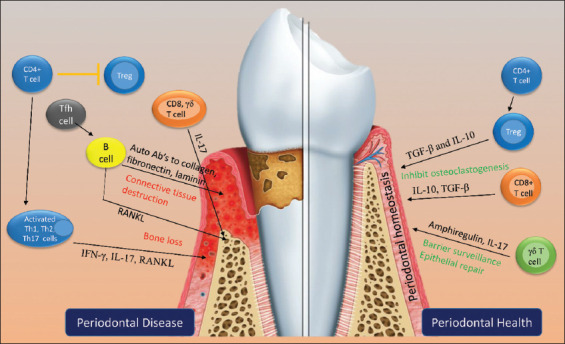
T cells in periodontal health and disease

The failure of complete resolution of inflammation by a weak Th1 response stimulates the mast cells to secrete IL-4, culminating in a Th2 response accompanied by B-cell activation with subsequent antibody production. The secretion of ineffective antibodies like IgG2 results in persistent inflammation, where chronic B-cell activity enhances IL-1 production eventually paving way to tissue destruction [52,60,61]. The presence of TGF-*β* coupled with IL-1/IL-6 due to the prevailing inflammatory state shifts the Tregs toward a Th17 response. Therefore, the periodontal microenvironment and costimulatory signals determine the plasticity of Th1/Th2 and Th17/Treg cells. Ultimately, the balance amongst the Th1/Th2/Th17/Treg cells is decisive in orchestrating a tissue-protective or tissue-destructive immuno-inflammatory response in the periodontium. These protective and destructive immune responses with the types of T cells involved in periodontal health and disease are depicted in Figure 2.

## 5. T Cells in Periodontal Disease

Subsequent to antigen presentation, the activated T-lymphocytes transform into any of the T-cell varieties influenced by the cytokine milieu in the local environment. The inflammation-enhancing cytokines such as IL-1, IL-17, and IL-25, which further activate DC, B cells, and neutrophils, are secreted by the triggered Th cell types 1, 2, and 17. Subsequent triggering of T- and B-lymphocytes due to persistent immune dysregulating reactions results in the production of RANKL instigating bone destruction around the tooth by disrupting the balance between osteoblasts and osteoclasts; the outcome of which is tooth loss. Osteoclastogenesis is regulated by wide cytokine network and complex inflammatory signals mediated through RANKL, IL-6, TNF-a, IL-1b, and PGE2 [62]. The enhanced expression of inflammation-promoting cytokines, such as TNF-a, IL-17, and IFN-g (Th1, Th2, and Th17 cells), surpasses the protective roles of IL-10 and TGF-b produced by Th2 and Tregs favoring osteoclastogenesis and bone loss [49]. The Tfh cells that activate B cells lead to clonal expansion as a result of which antibodies specific to the antigen are produced. However, it also results in the secretion of autoantibodies to host connective tissue proteins such as collagen, laminin, and fibronectin, which contribute to the local tissue destruction. In diseased conditions, the lack of Tregs or the insufficient regulatory actions to suppress the local inflammation by them also has a definite part in disease progression [2]. Increased gingival expression of Tregs in periodontitis patients has been documented by Cardoso *et al.*, where the Tregs could be engaged in modulating the local immune responses [63]. The cytokine IL-17 is critical in destruction of the alveolar bone around the tooth. In a study by Chen *et al.*, elevated tissue levels of Th1 and Th17 cells were present in periodontitis patients in contrast to their healthy controls, which were also correlated with probing depth [64]. The mRNA expression of IFN-g, IL-17, and T-bet also manifested a similar enhanced pattern [64]. The IL-17^+^ cells were characterized by Dutzan *et al*. who found that (i) Th cells are the major source, (ii) cells like CD8^+^ and gd T cells are the other minor sources, and (iii) the presence of IL-17^+^ cells dictates whether the periodontal disease is destructive or not [48,65]. It has been observed that periodontal cells in the diseased microenvironment, including fibroblasts, synergistically enhance the expression of genes related to osteoclastogenesis [66]. Periodontal bone loss can also occur in a RANKL-independent mode through a unique cytokine of T-cell named secreted osteoclastogenic factor of activated T cells. It coopts the osteoblasts and increases the osteoclastogenic cytokines which favor osteoclast activity [67]. Apart from autoantibodies from B cells, collagenases and other MMPs degrade the extracellular matrix and basement membrane components, resulting in periodontal tissue breakdown. In periodontitis, specific MMPs and their inhibitors are upregulated or suppressed by distinct cytokines. MMP-3, -8, and -9 secreted from gingival fibroblasts are upregulated by cytokines IL-1b, TNF-a and IL-6. MMP-1, -3 and -8 genes are suppressed by TGF-b, which upregulates MMP-2 and MMP-13 in the keratinocytes [68]. It has also been suggested that activated MMPs can further activate other MMPs through a mutual activation cascade [69]. MMP-8 predominantly released by neutrophils is a critical collagen-degrading enzyme in periodontitis which degrades interstitial collagens. Thus, an imbalance among the functionally diversified T-cell subsets further creates disparity in the osteoimmunology and connective tissue compartments, thereby disrupting the tissue homeostasis and shifting the balance towards periodontal destruction.

## 6. Therapeutic Approaches

At present, the treatment approaches for periodontal disease are mostly aimed at elimination of disease-causing pathogens. Considering the complex pathogenesis of periodontal diseases, various elements of the immune system involved could be potential therapeutic targets. The credible intervention aspects related to T-cell phenotype include (i) immunization with P.g which reduces bone loss, (ii) anti-IFN-*γ* therapy to counteract catastrophic Th1 cytokine activity, and (iii) administration of Tregs or anti-inflammatory molecules in polymeric carriers to counteract Th1 and Th17 actions, and guide tissue homeostasis and local DNA delivery of Th2 cytokines, which favors a shift toward Th2 phenotype resulting in stable periodontium [53]. Similarly, local administration of anti-TNF-*α*, RANKL inhibitors, PGE2 inhibitors, and MMP inhibitors that can indirectly hamper destructive T-cell actions by aiding in the prevention of bone and tissue destruction has also been suggested [70,71]. Therefore, therapeutic strategies modulating the immune and inflammatory responses could possibly restore the balance within the diseased periodontal environment, thereby preventing the detrimental consequence of immune response on periodontal tissues. Furthermore, evaluating the levels of specific cytokines of the destructive T cells such as IL-17 might help in monitoring periodontal disease progression and/or prognosticating therapeutic effects.

## 7. Future Perspectives

Overall, animal studies revealed the potential aspects of T-lymphocytes in the pathogenesis of periodontitis, whereas human studies showed conflicting findings which might be due to the variations in experimental approach and time of sampling relative to disease activity as periodontal disease is characterized by the on-and-off active and inactive periods. Moreover, since animal models cannot depict all aspects of periodontitis in humans, further studies that explore the complex underlying mechanisms of T-cell-mediated periodontal disease pathogenesis are necessary for identifying potential therapeutic targets. Based on therapeutic interventions, detailed studies on various T-cell types are needed to provide sufficient evidence for their application in humans health.

## 8. Conclusion

T-cell subsets are delineated by functional cytokine profiles rather than their phenotype. The main play of various classes of T-lymphocytes in the immunopathogenesis of periodontal disease at various levels is evident. Being a sovereign member, T cells are dynamic in nature, which is similar to that of periodontal disease. Ultimately, the balance amongst the Th1/Th2/Th17/Treg cells is decisive in orchestrating a tissue-protective or tissue-destructive immuno-inflammatory response in the periodontium. A sagacious understanding on the T-cell regulation of the intricate balance between the destructive and protective immune responses underscores the emergence of novel treatment approaches for periodontal diseases in the future. The currently available literature evidence, which is largely based on experimental periodontitis studies, requires cautious interpretation. Therefore, detailed studies are desired to provide commensurate evidence.
